# Behavioral assessment of auditory processing in adulthood: population of interest and tests - a systematic review

**DOI:** 10.1590/2317-1782/20232022044en

**Published:** 2023-05-01

**Authors:** Pamela Papile Lunardelo, Marisa Tomoe Hebihara Fukuda, Ana Cecília Grilli Fernandes Stefanelli, Sthella Zanchetta

**Affiliations:** 1 Faculdade de Filosofia, Ciências e Letras de Ribeirão Preto - FFCLRP, Universidade de São Paulo - USP - Ribeirão Preto (SP), Brasil.; 2 Faculdade de Medicina de Ribeirão Preto - FMRP, Universidade de São Paulo - USP - Ribeirão Preto (SP), Brasil.

**Keywords:** Adults, Middle Aged, Young Adult, Auditory Processing, Auditory Processing Disorder, ystematic Review

## Abstract

**Purpose:**

To identify the behavioral tests used to assess auditory processing throughout adulthood, focusing on the characteristics of the target population as an interest group.

**Research strategies:**

PubMed, CINAHL, Web of Science, and Scielo, databases were searched with descriptors: “auditory perception” or “auditory perception disorders” or “auditory processing” or “central auditory processing” or “auditory processing disorders” or “central auditory processing disorders” with adults OR aging.

**Selection criteria:**

Studies with humans included, the adult population from 18 to 64 years old, who performed at least one behavioral test to assess auditory processing in the absence of hearing loss.

**Data analysis:**

Data extraction was performed independently, using a protocol developed by the authors that included different topics, mainly the behavioral auditory tests performed and the results found.

**Results:**

Of the 867 records identified, 24 contained the information needed to answer the survey questions.

**Conclusion:**

Almost all studies were conducted verify performance in one or two auditory processing tests. The target target population was heterogeneous, with the most frequent persons with diabetes, stuttering, auditory processing disorder, and noise exposure. There is little information regarding benchmarks for testing in the respective age groups.

## INTRODUCTION

Central auditory processing (CAP) is responsible for the transformation, organization, decoding, and encoding of acoustic information over a short period of time. This action provides an effective and efficient analysis of verbal and nonverbal sounds by the central auditory nervous system (CANS)^(1)^. The neurobiological deficit that affects this system is called central auditory processing disorder (CAPD). This condition may be related to the impairment of neural connectivity of bottom-up and / or top-down pathways; for the latter, the regulatory effects of cognitive processes (e.g., memory, attention, and language) are involved^(2,3)^.

In the adult population with complaints about speech comprehension in a noisy environment, approximately 10% have hearing sensitivity within the normal range^(4,5)^. These findings characterize a unique clinical population but are not uncommon^(4,6-11)^. One of the reasons for this complaint is the presence of CAPD; although its prevalence is not well established for the adult population under the age of 60, where estimates vary between 0.5%, 14%, and 23%^(9,12,13)^.

CAPD results from different structural and functional etiological factors that affect the CANS or even in their absence^(1,14)^. A possible causal factor is the neural changes in the auditory pathways, which are independent of any type of peripheral hearing loss. These are attributed to the deterioration or decline of function throughout adult life before the cycle is understood as old age^(15)^. A decrease in the neural network in areas responsible for speech processing has been described in post-mortem studies carried out by Brody^(16)^. Even before 60 years of age, anatomical and physiological changes occur in the ventral cochlear nucleus, justifying the lower efficiency and accuracy of transmitting information in the CANS^(17)^. The interhemispheric function remains relatively stable until close to 40 years of age, with a decline from this age onwards. Men showed a change in function around age 35, whereas women maintained a stable performance until age 55^(18)^. Decreased estrogen levels in postmenopausal women may suppress the gamma-aminobutyric acid (GABA) inhibitor^(19)^, contributing to changes in CAP around the age of 50 years^(20)^. The decline of this inhibitor generates functional impairment, causing “neural noise, “which impairs speech perception. The decrease in GABA in the inferior colliculus as a function of increasing age was initially described in animals^(21)^; however, similar results were found in humans, which were related to the deterioration in the performance of speech recognition^(22)^.

The main focus of studies with CAP behavioral tests in young and middle-aged adults compared the auditory mechanisms as a function of a specific condition or pathology (e.g., diabetes mellitus, tinnitus, noise exposure, multiple sclerosis, stuttering, among others), usually with better performance by the healthy population^(23-35)^. The approach to increasing age has been less explored, especially in adults without hearing loss. Studies agree that young adults better understand speech in noise than older adults^(11,36-39)^ and even middle-aged adults in temporal processing^(40)^. A study with a population aged 50 to 70 years identified that the score on dichotic listening and temporal ordering tasks was only slightly lower than that expected for young adults. The authors inferred that if middle-aged adults were not included, the difference in performance between young and old adults would be greater^(41)^.

Changes in electrophysiological processing patterns during adulthood have also been documented. Reports of differences in the latency, amplitude, and quality of tracings at the brainstem, thalamus, and cortex levels have been described with increasing age^(42-46)^. A study showed that regardless of the auditory threshold, the amplitude of all auditory brainstem response (ABR) peaks decreases with advancing age, with an increase in the latency of waves I and III^(42)^. Another study found that between the ages 25 and 55, wave V latency increases by approximately 0.2 ms, while amplitude decreases by approximately 10%^(43)^. The frequency following response (FFR) wave amplitudes were also predominantly lower in older individuals^(44)^. Advancing age promotes an increase in the amplitude of the Na, Na-Pa, and Nb-Pb components of the middle latency auditory evoked potential (MLAEP), indicating a decrease in the capacity of the subcortical system to inhibit auditory responses^(45)^. Changes in auditory thalamocortical processes have also been reported in adults aged 19-45 years, with decreased P1 and N1 latencies throughout adulthood^(46)^. In the P300 component, there was a decrease in amplitude and an increase in latency. These changes occur at the same time as different cognitive declines, beginning around the age of 30^(47)^.

Another factor to be considered is the decline in cognitive functions, which, added to the impairment of auditory neural functions, can result in speech perception difficulties^(48)^. A decline in working memory has a negative effect on speech recognition in noise^(11,16)^. In environments where speech is degraded or competed with other acoustic stimuli, there is a greater perceptual demand and overload of this higher-order function^(49)^. Between the ages of 30 and 50, cognitive functions undergo continuous and monotonous decline, contributing to speech perception difficulties^(50)^.

The auditory system and areas of association undergo anatomical and physiological changes throughout life regardless of the type of pathology^(15,50)^. The harmful consequences of these changes should be the focus of future investigations in young and middle-aged adults. However, in different aspects, this population is underrepresented in the literature. The need to expand knowledge regarding CAPD assessment should be recognized. The basic principles of the choice of tests based on the population addressed^(51)^ and their sensitivity and specificity to identify CANS dysfunction^(1,2,14,52,53)^ need further consideration.

## PURPOSE

The present review aimed to identify the behavioral tests used to assess CAP throughout adulthood, focusing on the characteristics of the target population as an interest group. Additionally, aspects related to health conditions include, but are not limited to, occupational or leisure exposure to high sound intensities, test reference parameters, and the use of complementary assessments.

## RESEARCH STRATEGY

The present systematic review was carried out according to the Preferred Reporting Items for Systematic Reviews and Meta-Analyses (PRISMA) 2020 checklist^(54)^.

The search strategy was designed to identify potentially eligible records. The keywords were selected using the PubMed indexing vocabulary, Medical Subject Headings (MeSH Terms), and Health Science Descriptors library (DeCS) in English. From this, “auditory perception” or “auditory perception disorders” or “auditory processing” or “central auditory processing” or “auditory processing disorders” or “central auditory processing disorders” were combined with adults OR aging. The databases searched were PubMed (MEDLINE), CINAHL (EBSCO), Web of Science, and SciELO, which included the period (January 1, 2010, to July 30, 2021), age (18 to 64 years), humans, and study type (clinical study, clinical trial, multicenter study, observational study, randomized or uncontrolled trial).

## SELECTION CRITERIA

The selection of studies was performed by two reviewers (PPL and SZ) independently and blindly through the screening of records based on their titles and abstracts. Studies with humans selected for full reading: a) addressed the adult population aged 18 to 64 years (because some of the selected databases did not present this variable as a filter), b) performed at least one behavioral test for CAP assessment, and c) included populations without hearing loss of any type and degree. The full text was obtained from all records that met the eligibility criteria. In a disagreement between the two reviewers at any point in the selection process, a third reviewer (ACGFS) was consulted about the analysis.

## DATA ANALYSIS

The analysis of the articles was performed independently (ACGFS and PPL), and the collected data were compared. Initially, a pre-test was conducted with ten randomly selected articles to verify the occurrence of inaccuracies in the data extraction. The target information was distributed according to the different topics: a) basic data: year and date of publication; b) type of study; c) sample number; d) general age group and/or by groups; e) defined condition for constituting the groups and their eligibility criteria; f) criteria for defining hearing sensitivity; g) exposure to occupational noise; h) the processing tests performed and their respective mechanisms and abilities; i) standard of normality; and j) additional investigations: electrophysiological, electroacoustic, auditory self-perception, and mental state of consciousness.

To assess the quality of nonrandomized, case-control studies, the Newcastle-Ottawa Scale^(55)^ was used, which assesses aspects of group equality and the presence of bias. For observational studies, the Quality Assessment Tool for Observational Cohort and Cross-Sectional Studies^(56)^ was used.

## RESULTS

**Figure 1:**
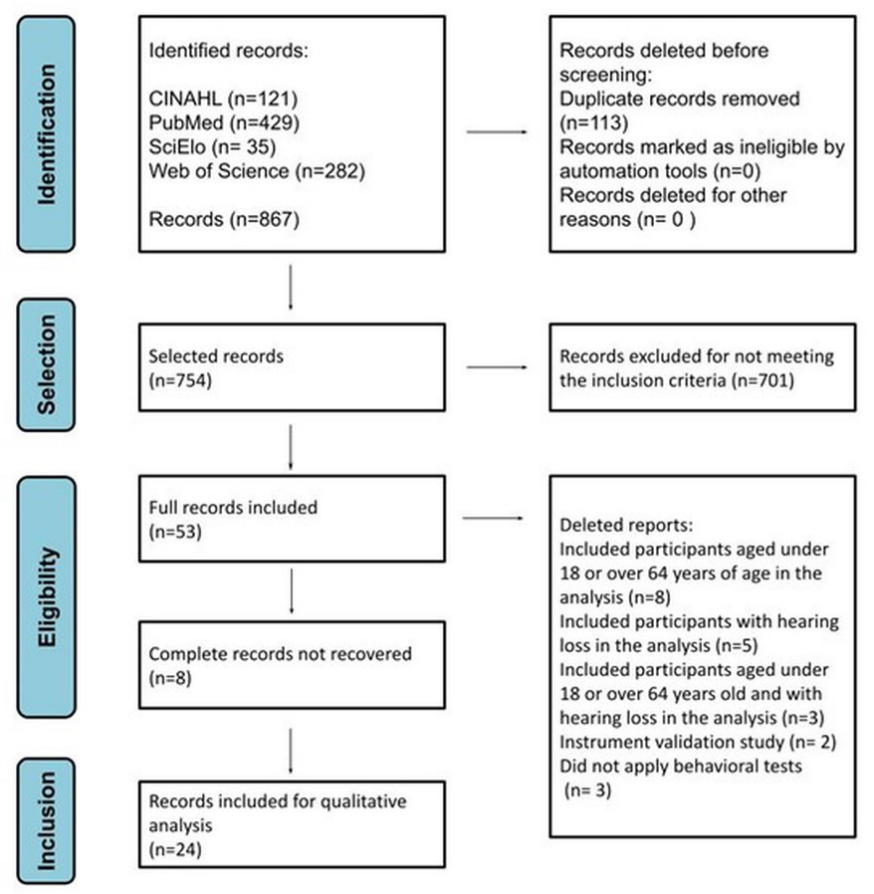
Systematic review steps (proposed PRISMA flowchart)

A total of 867 records were found, of which 53 were selected for reading in full, and 24 were classified as containing the information necessary to answer the research questions, corresponding to 2.7% (24/867) of the initial sample (Figure 1). The characteristics of the studies included in this review are presented in Table 1 in chronological order of publication.

**Table 1:** Characterization of the articles included in the review

**Author** (Year)		**Condition studied**	**Assessment behavioral**	**Main results of behavioral assessment of auditory processing**
		**Characterization of the population**		
**1**	Cameron et al.^(36)^	Healthy	LISN-S	● Worse performance of speech comprehension in noise for the group of older adults (30-60 years old) than the younger ones (18-30 years old);
		**N=**132		● Ability to use spatial cues does not diminish in individuals with normal hearing until age 60.
		Young: 36 (12-17 years)		
		Adults: 96 (18-60 years)		
**2**	O’Beirne et al.^(37)^	Healthy	LPFST	● There was no difference between the scores obtained in the RE and LE in both groups;
		**N**=63		● Improvement in performance with increasing age between 17 and 34 years, decline in speech understanding from 35 years of age.
		Adults= 15 (28.5 years)		
		Children= 15 (10.1 years)		
**3**	Liberalesso et al.^(57)^	Sleep Deprivation	SSW, RGDT	● Worse performance on RGDT and SSW after 24 hours of deprivation
		**N**=90 (18-40 years)		of sleep;
				● No sex effect on SSW and RGDT.
**4**	Sininger et al.^(35)^	Hearing lateralization	Discrimination of intensity, frequency and temporal resolution	● Lower threshold for detection of silent interval in LE;
		**N**=34 (18-32 years)		● LE advantage for tonal stimulus, no advantage for noise;
		**Education:** Average - 15.69 years		● Difference between RE and LE for intensity discrimination decreased with age.
		**Musical training:** Average - 2.7 years		
**5**	Iliadou et al.^(28)^	Psychosis	GIN, RGDT	● No difference between the Psychosis and Musicians group in the GIN test;
		**N**=90		●Psychosis Group with better performance in the GIN in relation to the RGDT;
		Psychosis= 17 (18-48 years)		● Best performance for the Musicians group in the RGDT.
		Musicians= 11 (28-61 years)		
**6**	Saunders et al.^(58)^	War veterans exposed to blast	HINT, LISN-S, ATTR, TCST, SSW	● 75% reported having difficulty understanding speech in noise;
		**N**=99 (25-53 years)		● 56.6% found it difficult to follow conversations;
		**Complaint:** 80% migraine; 73% Dizziness		● 60% showed alterations in the HINT and 33.7% in the SSW.
		**Health condition:** 19% PTSD		
		**Education:** High School - Undergraduate		
**7**	Prestes et al.^(29)^	Stutter	DPS, RGDT	● Stuttering group with better performance in DPS and RGDT in relation to non-stutterers, with values below normality.
		**N**=41		
		Control= 21 (18-46 years)		
		Study= 20 (18-46 years)		
**8**	Kumar et al.^(39)^	Healthy	TFC, SPIN	● Younger adults performed better on both tests;
		**N**=29		● Worse performance of young and older adults with increasing comprehension rate and/or signal-to-noise ratio.
		Control= 15 (18-25 years)		
		Study= 14 (30-50 years)		
**9**	Przewoźny et al.^(59)^	Arterial hypertension	MAA, RGDT	● Arterial hypertension group with higher silent interval detection thresholds, but with no significant difference between the groups;
		**N**=64		● Arterial hypertension group with worse sound localization performance.
		Control= 32 (52.8 years)		
		Study= 32 (53.1 years)		
		**Health condition:** Pharmacological treatment; Incidence of hyperlipidemia; smoking.		
**10**	Santiago et al.^(60)^	Healthy	MLD	● Positive correlation between MLD and waves V, A and F of the FFR;
		**N**=20 (18-30 years)		● The higher the latency of waves V, A and F, the higher the MLD.
**11**	Roup et al.^(61)^	Hearing difficulty	SCAN-3:A, MLD	● All individuals with complaints showed altered performance in at least one of the behavioral tests;
		**N=**37	GIN, DDT, SPIN	● 12% showed alterations in MLD and SCAN-A:3, 41% in DDT, 53% in GIN, 71% to 88%.
		Study= 20 (19-27 years)		
		Control= 17 (18-58 years)		
**12**	Gallun et al.^(62)^	War veterans exposed to blast	GIN, DDT, PPS, SSW, MLD	● War veterans and control group with the worst RE performance in the GIN;
		**N**=59		● War veterans with the worst performance in DDT, SSW, PPS and MLD.
		Control= 29 (39.2 years)		
		Study= 30 (37.3 years)		
		**Health condition:** 56.7% PTSD		
**13**	Mishra et al.^(24)^	Diabetes Mellitus Type 2	GDT	● Diabetes group with the highest silent interval detection threshold;
		**N**=30		● Mean threshold in GDT: GE= 6.49 ms (0.81); GC=3.33 ms (0.79).
		Control= 15 (30-40 years)		
		Study= 15 (30-40 years)		
**14**	Ibraheem et al.^(23)^	Tinnitus	GIN	● Tinnitus group with worse performance in the GIN test;
		**N**=30		● No correlation between GIN and tinnitus duration, subjective scale, audiological profile and psychoacoustic measures of tinnitus;
		Control= 15 (20-45 years)		● Positive correlation between OAE amplitude and GIN scores.
		Study= 15 (20-45 years)		
**15**	Silva et al.^(33)^	Diabetes Mellitus Type 1	List of Sentences in Portuguese	● Significant differences between the groups with and without diabetes for the recognition threshold in silence, in noise and in the signal-to-noise ratio.
		**N=**40		
		Control=20 (18-30 years)		
		Study=20 (18-30 years)		
**16**	Hoover et al.^(34)^	Mild Traumatic Brain Injury	QuickSIN, SRM	●Presence of auditory handicap increases the probability of worse speech performance in noise;
		**N**=33		●No difference in speech comprehension performance in subjects with and without mild traumatic brain injury.
		Control= 9 (18-24 years)		
		Study= 13 (25-71 years)		
		Paired= 11 (20-70 years)		
**17**	Arcuri et al.^(27)^	Stutter	TFR, TDNV, SSW, DPS, PPS, SSI, RGDT	●Stuttering group with the worst performance in the TDNV and PPS tests;
		**N**=30		●14 participants of the Stuttering Group presented alterations in the CAPD.
		Control= 15 (18-40 years)		
		Study= 15 (18-40 years)		
**18**	Yeend et al.^(31)^	Noise exposure	LISN-S, NALDCT, TFS, AM	●No correlation between lifetime noise exposure and performance of auditory processing tasks;
		**N**=122 (30-60 years)		●Positive correlation between speech comprehension in noise and working memory, attention, high-frequency tonal thresholds and suppression strength of the medial olivocochlear system.
		**Complaint:** Tinnitus; difficulty understanding speech in noise; discomfort for loud sounds		
		**Education:** 68% Graduates; 25% Technical qualification; 6% High School		
		**Health conditions:** Smoking; use of ototoxic; otitis history		
		**Musical training:** 18% ≤ 8 years; 40% ≥ 8 years; 17% professionals; 25% no experience		
		**Noise exposure:** 70% occupational		
**19**	Fostick et al.^(32)^	Dyslexia	Judgment of temporal order	●Dyslexia group with worse performance in temporal processing;
		**N=**101		●Positive correlation between working memory performance and temporal processing with reading and phonological processing.
		Control= 23 (20-33 years)		
		Study= 78 (20-33 years)		
		**Education:** 13 to 15 years		
**20**	Habibi et al.^(25)^	Multiple Sclerosis	SSW persian version, DDT	●46% of the multiple sclerosis group showed alterations in the SSW;
		**N**=90		●Multiple Sclerosis group with higher percentage of qualitative and quantitative errors in SSW and worse performance in DDT.
		Control= 45 (25-45 years)		
		Study= 45 (25-45 years)		
		**Multiple Sclerosis=** 04 to 10 years		
**21**	Trott et al.^(20)^	Post-menopause	DDT, DPS, LINS-S, SPIN-R	●No difference between pre- and post-menopausal women for DDT, DPS and SPIN-R;
		**N**=28 (18-70 years)		●Worse performance of the postmenopausal group on LISN-S.
		Control= 14		
		Study= 14		
**22**	Sanguebuche et al.^(63)^	CAPD	DSI, MLD, PPS, DPS, RGDT, TFCA	●18 to 29 years: Better scores for the CG, except in the RE of the DSI and in both ears of the TFCA;
		**N**=94		●30 to 58 years: Best scores for the CG in the DSI (LE), RGDT and TFCA (RE).
		Control= 64 (18-59 years)		
		Study= 30 (18-59 years)		
		**Education:** Minimum of 11 years		
**23**	Turcatto et al.^(30)^	CAPD	DDT, PPS, TFC	●Better performance of the group without CAPD in the PPS;
		**N**=40		●Similar performance between individuals with and without CAPD in DDT;
		Control= 20 (18-35 years)		●Association between scores on the self-perception scale and the
		Study = 20 (18-35 years)		list of monosyllables in the TFC.
		**Education:** 95% incomplete higher education		
**24**	Pham et al.^(64)^	Nicotine	GDT	●Lower silent interval detection threshold with nicotine use;
		**N**=14 (18-27 years)		●Better performance of selective attention with nicotine use.
		**Health conditions:** Oxygenation monitoring		

**Caption:** LINS-S = Listening in Spatialized Noise-Sentences; LPFST = Low-pass Filtered Speech Test; CAPD = Central Auditory Processing Disorder; RE = Right Ear; LE = Left Ear; SSW = Staggered Spondaic Word Test; PTSD = Post Traumatic Stress Disorder; RGDT = Random Gap Detection Test; ms = milliseconds; GIN = Gap in Noise Test; HINT = Hearing in Noise Test; ATTR = Adaptive Tests of Temporal Resolution; TCST = Time Compressed Speech Test; LLAEP = Long Latency Auditory Evoked Potential; DPS = Duration Pattern Sequence; TFC = Compressed Speech Test; SPIN = Speech Perception in Noise Test; PASN = Sensorineural Hearing Loss; CG = Control Group; EG = Study Group; MAA = Horizontal Minimum Audible Angle Test; MLD = Masking Level Difference; ABR = Auditory Brainstem Response; FFR = Frequency Following Response; SCAN A = Tests for Auditory Processing Disorders in Adolescents and Adults; DDT = Dichotic Digit Test; PPS = Pitch Pattern Sequence; GDT = Gap Detection Threshold; QuickSIN = Quick Speech in Noise; SRM = Spatial Release from Masking; OAE = Otoacoustic Emission; TFR = Speech-in-Noise Test; TDNV = Non-verbal Dichotic Test; NALDCT = National Acoustic Laboratories Dynamic Conversations Test; TFS = Temporal Fine Structure; AM = Amplitude Modulation; SSI = Synthetic Sentence Identification; DSI = Identification of Dichotic Sentences; TFCA = Adapted Compressed Speech Test

### Characteristics of the studies: type, population, and quality of the study

In the analysis of the type of study, 70.8% (17/24) were of the case-control type, and 29.2% (7/24) were of the observational type.

The case-control studies (17/24) evaluated different populations, with two studies each (11.7%) covering: diabetes mellitus (studies 13 and 15), stuttering (studies 07 and 17), and CAPD (studies 22 and 23). Other conditions which each addressed a single study (5.9%) were sleep deprivation (study 3), psychosis (study 5), arterial hypertension (study 9), speech comprehension complaint (study 11), tinnitus (study 14), mild traumatic brain injury (study 16), noise exposure (study 18), dyslexia (study 19), multiple sclerosis (study 20), post-menopause (study 21), and nicotine exposure (study 24).

Of the observational studies (7/24), 43.9% (3/7) investigated performance between different ages (studies 01, 02, and 08), 28.6% (2/7) covered a population with a history of exposure to noise (studies 06 and 18). The other two investigations, each 14.3% (1/7), addressed hearing lateralization (study 04) and the correlation between hearing tests (study 10).

Based on the Newcastle-Ottawa Scale criteria, 82.3% (14/17) of the studies (3, 7, 9, 11, 12, 13, 14, 15, 17, 20, 21, 22, 23, and 24) obtained a classification higher than six, indicating the quality of the studies. For observational studies, in the analysis using the Quality Assessment Tool for Observational Cohort and Cross-Sectional Studies, 100% had a score that indicated good quality (studies 1, 2, 4, 6, 8, 10, 18).

### Performance differences in behavioral tests depending on the condition

Among the conditions evaluated in the case-control studies, 94.1% (16/17) identified that the “case group” presented a worse performance in one or more auditory skills in relation to the control group. In studies in which more than one auditory ability was investigated, some of them differentiated the groups: sound localization for arterial hypertension (study 9); dichotic listening, auditory closure, and temporal resolution in CAPD (studies 22 and 23); non-verbal background figure and temporal ordering in stuttering (study 17); temporal resolution in psychosis (study 5); and postmenopausal auditory closure (study 21). For the mild traumatic brain injury condition (study 16), there was no difference between the groups, representing 5.9% (1/17) of the studies.

The criteria that led to the constitution of the “case group” are diverse and reflect conditions and/or characteristics that have already been described as etiological or comorbid factors for CAPD. This fact is justified because there is an etiological heterogeneity for CAPD^(1)^, which has been documented in cases of chronic metabolic^(24,33)^, vascular^(59)^, demyelinating^(25)^, hormonal^(20)^, psychiatric^(28)^, sleep^(57)^, learning^(32)^, and fluency disorders^(27,29)^.

In all of the conditions reported above, performance in auditory skills was lower in the case group. This is particularly true for temporal and auditory closure skills, validating the importance of assisting these populations. It is necessary to emphasize that the two conditions addressed do not start in adulthood. Dyslexia^(32)^ and developmental stuttering^(27,29,32)^ are conditions present since childhood, and the relationship established with CAP negatively affects these individuals throughout their lives^(27,29,32)^.

Another important consideration of the conditions studied is that exposure to noise is the most explored^(31,58,62)^. One of its harmful effects is the damage in cortical areas responsible for CAP, which manifests as a speech comprehension complaint without alteration of the auditory thresholds^(31,59)^. Establishing a relationship between noise exposure and auditory closure ability is a complex task because of the influence of supramodal factors on hearing^(31)^. However, regardless of this, it is known that this population performs below expectations without spontaneous improvement even after years of exposure to loud noises^(31,58,62)^. Finally, it is worth noting the conditions of nicotine exposure^(64)^. This was the only study that investigated the possibility of treatment based on the hypothesis that this substance would increase auditory gating function in adverse listening situations. The manipulated use of nicotine favors selective attention and can be used in young adults with acetylcholinergic deficits^(64)^.

### Performance differences in behavioral tests as a function of age

Only 12.5% (3/24) of the studies (1, 2, and 8) measured differences in performance on CAP behavioral tests throughout adulthood. All of them evaluated auditory closure ability with different tests, namely the Listening in Spatialized Noise-Sentences (Study 1), low-pass filtered speech test (Study 2), time-compressed speech test, and speech perception in noise (Study 8). Three studies identified that older adults performed worse than younger adults. Specifically, Study 1 identified that the performance of speech understanding in noise by adults aged 30-60 years was lower than that of adults aged 18-30 years. Study 2 found that auditory closure ability improves up to 34 years of age and declines from this age onwards. Study 8 identified that adults aged 18 to 25 years presented a better performance in the two tests applied compared to adults aged 30 to 50 years.

From these results, it is evident that few studies have investigated CAP in relation to the changes inherent in the increase in age in adulthood^(36,37,39)^. All of them evaluated only auditory closure ability. Regardless of the type of stimulus used, words, or phrases, the findings between the studies were similar, indicating that adults under 60 years of age performed worse than young adults. From these studies, it can be inferred that adults over 30 years of age experience disadvantages in adverse listening conditions, even if their ability to analyze acoustic cues from sound stimuli does not suffer this decline^(36,37,39)^.

This knowledge supports the importance of investigating auditory disorders at the CANS level in the healthy adult population, including all auditory skills, and comparing groups with less variation in age. This would allow an understanding of this dynamic process of increasing age, both in terms of function and time of onset.

Although it is challenging to identify the point at which the decline in CAP begins in adult life, this investigation is necessary for each of the mechanisms and skills. Since timely information processing is essential for communication, deceleration related to age is well documented in the cognitive and sensory domains.

### Characterization of the studied population

A single study (4.2%) characterized the investigated population in terms of independent variables, health status, and exposure to occupational and leisure noise (study 18). Health conditions were characterized by using potentially ototoxic drugs, contact with ototoxics in general, smoking history, ear infection, and tinnitus. Other studies performed some types of characterization but did not analyze them as independent variables for the conditions investigated.

### Behavioral investigation of auditory processing

From the 24 studies included, it was possible to identify more than 20 variations in the behavioral tests. Of these, the test most applied was the Random Gap Detection Test (RGDT), present in 25% (6/24) of the studies, followed by the Pitch Pattern Sequence (PPS), Digit Dichotic Tests (DDT), and Staggered Spondaic Word (SSW) used in 20.8% (5/24) of the studies. Listening in spatialized noise sentences, gap in noise, duration pattern, and masking level difference tests were performed in 16.6% (4/24) of the studies. Other tests were conducted in three or fewer studies.

Among the auditory skills evaluated, auditory closure was the most investigated (54.1%; 13/24), followed by resolution skills (45.8%, 11/24) and temporal ordering (41.6%; 10 /24). Verbal figure-ground and binaural interaction skills were assessed in 29.1% (7/24) of the studies, and non-verbal figure-ground skills in 4.1% (1/24). Only 20.8% (5/24) of the studies evaluated a single auditory ability, 12.5% (3/24) evaluated temporal resolution and auditory closure, and 4.1% evaluated binaural interaction and verbal figure-ground.

Although the tests that appeared in a greater number of articles were the RGDT, PPS, DDT, and SSW, the low redundancy tests were identified with greater diversity, and more than 10 tests were intended to assess auditory closure ability. This finding needs to be discussed, as it is the most investigated auditory skill. This is possibly because it is intrinsically related to speech comprehension^(14,52,53)^, and regarding a large number of tests found, probably because of the tests’ necessary characteristics. These must be validated in the language of the evaluated population, and recording parameters such as frequency, resonance, vocal modulation, articulation, and speech rate must be as adequate and natural as possible^(37)^. One should also consider the choice of the speech material and intrinsic redundancies, whether due to competitive stimuli or stimulus degradation, and the location of the sound source. These characteristics make the development and choice of these tests challenging, as the attempt is to get as close as possible to the adverse listening situations present in everyday life^(37,65)^.

### Determining the condition of auditory ability and auditory processing

Regarding normative values, 33.3% (8/24) indicated the use of references intended for the adult population to classify performance in the behavioral tests as adequate or altered (Studies 3, 7, 9, 11, 16, 17, 22, and 23). Two (8.3%) of the 24 identified studies aimed to determine the presence of CAPD (studies 22 and 23), for which the criteria used were alterations in one or more auditory skills^(52)^ (study 22) and alterations in the dichotic tests of digits and/or frequency pattern (study 23).

The application and interpretation of tests according to production and/or standardization recommendations reduces the variability of the interpretations and increases clinical consensus regarding results^(66)^. The diagnosis of CAPD was the objective of two studies; however, only one of them was undertaken as recommended by experts in the field^(63)^. It is well established in the literature that the diagnostic evaluation of CAPD should be performed through different behavioral tests that are sensitive and specific to identify CANS dysfunction^(1,2,14,52,53)^.

### Complementary investigation

Complementary tests were applied to the CAP assessment in 58.3% (14/24) of the studies. Of these 14 studies, 28.6% (4/14) applied auditory electrophysiological tests with 21.4% (3/14) including the click ABR (studies 10, 21, and 22), and 7.1% applied the FFR (study 10), MLAEP (study 21), and LLAEP (study 7). The use of otoacoustic emissions occurred in 35.7% (5/14) of the studies, varying between distortion products (studies 4, 9, and 18) and transients (studies 14 and 17). Regarding self-perception, 50% (7/24) of the studies used questionnaires to characterize the participants' perception of auditory function (studies 6, 11, 12, 14, 16, 18, and 23). The state of mental consciousness, in the form of screening and assessment, was investigated in only 21.4% (3/24) of the studies (6, 12 and 18).

Complementary tests can help diagnose CAPD as well as in the delimitation of this typically heterogeneous population^(1,2)^. However, the present review identified that this is not a common practice in studies including adults. Self-perception questionnaires were the most applied form of complementary assessment, possibly because some questionnaires showed a significant correlation with the findings of auditory behavioral tests^(1,2,53)^. Electrophysiological and electroacoustic tests have been applied in several studies. The literature recommends that these be included in CAP assessments. They allow the assessment of the functional and structural integrity of the auditory pathway and expand the understanding of the findings of behavioral tests^(1,14,53)^. Finally, mental status screening was the least performed complementary assessment, which ensured that the CAP findings were not consequences of significant cognitive changes and excluded this predictor factor. Therefore, it is worth reflecting that these factors that delimit the population and help in the diagnosis should be used because of the heterogeneity of CAPD and the influence of supramodal factors on hearing in the behavioral assessment.

## CONCLUSION

Most eligible studies aimed to evaluate a specific auditory mechanism and/or task in specific populations, not the diagnosis of CAPD itself. The most commonly used test was the RGDT, while auditory closure ability was the most investigated, with the greatest diversity of tests. Heterogeneity was also identified in the studied population regarding the characteristics of the case groups. Complementary assessment forms included electrophysiological and electroacoustic tests, self-perception questionnaires, and mental status screenings.
