# Supplementary material for: Behavioral assessment of auditory processing in adulthood: population of interest and tests - a systematic review
Source: Codas. 2023 May 1;35(2):e20220044. doi: 10.1590/2317-1782/20232022044en (PMC10162648; doi:10.1590/2317-1782/20232022044en)
Supplement: Translation

# Translation: Avaliação comportamental do processamento auditivo na idade adulta: população de interesse e testes - uma revisão sistemática

**DOI:** 10.1590/2317-1782/20232022044pt

**Authors:** Pamela Papile Lunardelo, Marisa Tomoe Hebihara Fukuda, Ana Cecília Grilli Fernandes Stefanelli, Sthella Zanchetta

**Affiliations:** 1 Faculdade de Filosofia, Ciências e Letras de Ribeirão Preto - FFCLRP, Universidade de São Paulo - USP - Ribeirão Preto (SP), Brasil.; 2 Faculdade de Medicina de Ribeirão Preto - FMRP, Universidade de São Paulo - USP - Ribeirão Preto (SP), Brasil.

**Keywords:** Adultos, Meia Idade, Adulto Jovem, Processamento Auditivo, Transtornos da Percepção Auditiva, Revisão Sistemática

## Abstract

**Objetivo:**

Identificar os testes comportamentais utilizados para a avaliação do processamento auditivo ao longo da vida adulta, com enfoque nas características da população alvo enquanto grupo de interesse.

**Estratégia de pesquisa:**

As bases de dados consultadas foram o *PubMed*, CINAHL, *Web of Science* e *Scielo*, a partir dos descritores: *“auditory perception” or “auditory perception disorders” or “auditory processing” or “central auditory processing” or “auditory processing disorders” or “central auditory processing disorders”* com *adults OR aging*.

**Critérios de seleção:**

Incluiu-se estudos com humanos, que abordaram a população adulta de 18 a 64 anos, que realizaram pelo menos um teste comportamental para avaliação do processamento auditivo, na ausência de perda auditiva.

**Análise dos dados:**

Realizou-se a extração de dados de forma independente, a partir de um protocolo desenvolvido pelos autores incluindo diferentes tópicos, principalmente os testes auditivos comportamentais realizados e os resultados encontrados.

**Resultados:**

Dos 867 registros identificados, 24 foram selecionados como contendo as informações necessárias para responder às perguntas de pesquisa.

**Conclusão:**

Quase a totalidade dos estudos foi conduzida com objetivo de verificar o desempenho em um ou dois testes de processamento auditivo; a população alvo foi heterogênea, as mais frequentes foram diabetes, gagueira, transtorno do processamento auditivo e exposição ao ruído. Há poucas informações sobre os padrões de referência para os testes na respectiva faixa etária.

## INTRODUÇÃO

O Processamento Auditivo Central (PAC) é responsável pela transformação, organização, decodificação e codificação das informações acústicas em um breve período de tempo, ação que propicia uma análise eficaz e eficiente dos sons verbais e não verbais pelo Sistema Nervoso Auditivo Central (SNAC)^(1)^. O déficit neurobiológico que acomete este sistema é denominado como Transtorno do Processamento Auditivo Central (TPAC). Esta condição pode estar relacionada ao comprometimento da conectividade neural das vias *bottom-up* e/ou *top down*, nesta última ocorre o envolvimento dos efeitos regulatórios de processos cognitivos (e.g. memória, atenção e linguagem)^(2,3)^.

Na população adulta com queixa de compreensão de fala no ruído, cerca de 10% possui sensibilidade auditiva dentro dos padrões de normalidade^(4,5)^, esses achados caracterizam uma população clínica única, mas não incomum^(4,6-11)^. Uma das justificativas para a referida queixa é a presença de TPAC, ainda que sua prevalência não esteja bem estabelecida para a população adulta com idade inferior a 60 anos, as estimativas variam entre 0,5%, 14% e 23%^(9,12,13)^.

O TPAC decorre de diferentes fatores etiológicos estruturais e funcionais que acometem o SNAC ou até mesmo na ausência deles^(1,14)^. Um possível fator causal são as mudanças neurais das vias auditivas, que independem de qualquer tipo de perda auditiva periférica, estas são atribuídas à deterioração ou declínio da função ao longo da vida adulta, antes do ciclo compreendido como velhice^(15)^. A diminuição da rede neuronal em áreas responsáveis pelo processamento da fala foi descrita em estudos *post-mortem* realizados por Brody^(16)^. Antes mesmo dos 60 anos de idade ocorrem alterações anatômicas e fisiológicas do núcleo coclear ventral, justificando a menor eficiência e acurácia na transmissão de informações no SNAC^(17)^. A função inter-hemisférica permanece relativamente estável até próximo aos 40 anos, com declínio a partir desta idade. Os homens apresentam alteração desta função próximo aos 35 anos, enquanto as mulheres mantêm o desempenho estável até os 55 anos de idade^(18)^. A diminuição do estrogênio em mulheres após a menopausa pode suprimir o inibidor ácido gama-aminobutírico (GABA)^(19)^, contribuindo para alterações do PAC por volta dos 50 anos^(20)^. O declínio deste inibidor gera um comprometimento funcional, resultando em “ruído neural” que prejudicaria a percepção de fala. A diminuição do GABA no colículo inferior em função do aumento da idade foi descrita inicialmente em animais^(21)^, resultados semelhantes foram encontrados em humanos, correlacionados ao pior desempenho no reconhecimento de fala^(22)^.

O principal enfoque dos estudos com testes comportamentais do PAC em adultos jovens e de meia idade comparam os mecanismos auditivos em função de uma condição ou patologia específica (e.g. diabetes mellitus, zumbido, exposição a ruído, esclerose múltipla, gagueira, entre outros) usualmente com melhor desempenho para a população hígida^(23-35)^. O enfoque do aumento da idade é menos explorado, principalmente em adultos sem perda auditiva. Os estudos concordam que jovens adultos apresentam melhor desempenho na compreensão de fala no ruído que adultos mais velhos^(11,36-39)^ e até mesmo adultos de meia idade no processamento temporal^(40)^. Um estudo com a população de 50 a 70 anos identificou que a pontuação nas tarefas de escuta dicótica e ordenação temporal foi apenas ligeiramente inferior ao esperado para jovens adultos, os autores inferem que se não fosse incluído os adultos de meia idade a diferença de desempenho entre jovens e idosos seria maior^(41)^.

As mudanças nos padrões do processamento eletrofisiológico na vida adulta também são documentadas. O relato de diferenças na latência, na amplitude e na qualidade dos traçados em nível de tronco encefálico, tálamo e córtex foram descritas com o aumento da idade^(42-46)^. Um estudo demonstrou que independente do limiar auditivo, a amplitude de todos os picos do Potencial Evocado Auditivo de Tronco Encefálico (PEATE) diminuem com o avanço da idade, com aumento da latência das ondas I e III^(42)^. Outro estudo encontrou que entre as idades de 25 a 55 anos a latência da onda V aumenta cerca de 0,2 ms, enquanto a amplitude diminui em cerca de 10%^(43)^. No *Frequency Following Response* (FFR) as amplitudes das ondas também foram predominantemente menores no grupo de indivíduos mais velhos^(44)^. O avanço da idade promoveu aumento na amplitude das componentes Na, Na-Pa e Nb-Pb do Potencial Evocado Auditivo de Média Latência (PEAML), indicando diminuição da capacidade do sistema subcortical em inibir respostas auditivas^(45)^. Mudanças nos processos talámo-corticais auditivos também foram relatadas em adultos com idades entre 19 e 45 anos, com diminuição das latências de P1 e N1 ao longo da vida adulta^(46)^. Na componente P300 há diminuição da amplitude com aumento de latência. Estas mudanças ocorrem no mesmo momento que diferentes declínios cognitivos, com início próximo aos 30 anos de idade^(47)^.

Outro fator a ser considerado é o declínio das funções cognitivas, que somado ao comprometimento das funções neurais auditivas pode resultar em dificuldades de percepção de fala^(48)^. O declínio da memória de trabalho exerce efeito nocivo no reconhecimento de fala no ruído^(11,16)^. Em ambientes em que a fala está degradada ou em competição a outros estímulos acústicos, há maior demanda perceptual e sobrecarga desta função de ordem superior^(49)^. Entre os 30 e 50 anos as funções cognitivas entram em declínio contínuo e monótono, contribuindo para as dificuldades de percepção de fala^(50)^.

O sistema auditivo e as áreas de associação sofrem alterações anatômicas e fisiológicas ao longo da vida, independente de qualquer tipo de patologia^(15,50)^. As consequências nocivas destas alterações deveriam colocar os adultos jovens e de meia idade como foco de investigação, entretanto em diferentes âmbitos esta é uma população sub-representada pela literatura. Deve-se reconhecer a necessidade de ampliar os conhecimentos quanto à avaliação do TPAC. Considerando os princípios básicos da escolha dos testes pautada na população abordada^(51)^ e na sensibilidade e especificidade destes para identificar as disfunções do SNAC^(1,2,14,52,53)^.

## OBJETIVO

A presente revisão tem como objetivo identificar os testes comportamentais utilizados para a avaliação do PAC ao longo da vida adulta, com enfoque nas características da população alvo enquanto grupo de interesse. Assim como, os aspectos relacionados às condições de saúde, entre eles, mas não somente, a exposição ocupacional ou de lazer a intensidades sonoras elevadas, os parâmetros de referência dos testes e o uso de avaliações complementares.

## ESTRATÉGIA DE PESQUISA

A redação da presente revisão sistemática foi realizada de acordo com os itens dos checklists *Preferred Reporting Items for Systematic Reviews and Meta-Analyses* (PRISMA) 2020^(54)^.

A estratégia de busca foi delineada a fim de identificar os registros potencialmente elegíveis. A seleção dos unitermos foi realizada a partir do vocabulário de indexação da PubMed, *Medical Subject Headings* (MeSH Terms) e na biblioteca de Descritores em Ciência da Saúde (DeCS), no idioma inglês. A partir disto combinou-se “*auditory perception” or “auditory perception disorders” or “auditory processing” or “central auditory processing” or “auditory processing disorders” or “central auditory processing disorders*” com *adults* OR *aging*. As bases de dados pesquisadas foram a Pubmed (MEDLINE), CINAHL (EBSCO), Web of Science e Scielo, os filtros selecionados foram o período (01 de janeiro de 2010 até 30 julho de 2021), idade (18 a 64 anos), humanos e tipo do estudo (estudo clínico, ensaio clínico, estudo multicêntrico, estudo observacional, ensaio randomizado ou não controlado).

## CRITÉRIOS DE SELEÇÃO

A seleção dos estudos foi realizada por dois revisores (PPL e SZ) de forma independente e cega, por meio da triagem dos registros a partir de seu título e resumo. Foram selecionados para leitura na íntegra os estudos com humanos que a) abordaram a população adulta, de 18 a 64 anos (em função de algumas das bases de dados selecionadas não apresentarem essa variável como filtro), b) realizaram pelo menos um teste comportamental para avaliação do PAC, c) populações sem perda auditiva, de qualquer tipo e grau. O texto completo foi obtido para todos os registros que atenderam aos critérios de elegibilidade. Na presença de discordância entre os dois revisores em algum momento no processo de seleção, um terceiro (ACGFS) revisor seria consultado para a análise.

## ANÁLISE DOS DADOS

A análise dos artigos foi conduzida de forma independente (ACGFS e PPL) e, posteriormente, os dados coletados foram confrontados. Inicialmente conduziu-se um pré-teste com 10 artigos selecionados aleatoriamente, a fim de verificar a ocorrência de imprecisões na extração dos dados. As informações alvo foram distribuídas em diferentes tópicos: a) dados básicos: ano e data da publicação; b) tipo de estudo; c) número amostral; d) faixa etária geral e/ou por grupos; e) condição definida para constituição dos grupos e seus critérios de elegibilidade; f) critérios para definição da sensibilidade auditiva; g) exposição ao ruído ocupacional; h) os testes de processamento realizados e seus respectivos mecanismos e habilidades; i) padrão de normalidade; j) investigações adicionais: eletrofisiológica, eletroacústica, auto-percepção auditiva e estado de consciência mental.

Para a avaliação da qualidade dos estudos do tipo caso controle, não randomizado, utilizou-se a *Newcastle-Ottawa Scale* ^(55)^ que avalia os aspectos de equiparação dos grupos e presença de viés; para os estudos do tipo observacional selecionou-se o *Quality Assessment Tool for Observational Cohort and Cross-Sectional Studies* ^(56)^.

## RESULTADOS

A partir da busca foram encontrados 867 registros, dos quais 53 foram selecionados para leitura na íntegra e 24 foram classificados como contendo as informações necessárias para responder às perguntas de pesquisa, correspondendo a 2,7% (24/867) da amostra inicial (Figura 1). As características dos estudos incluídos nesta revisão estão apresentadas na Tabela 1, em ordem cronológica de publicação.

**Figura 1:** Etapas da revisão sistemática (fluxograma proposto PRISMA)

**Tabela 1:** Caracterização dos artigos incluídos na revisão

**Autor** *(Ano)*		**Condição estudada**	**Avaliação comportamental**	**Principais resultados da avaliação comportamental do processamento auditivo**
		**Caracterização da população**		
**1**	Cameron et al.^(36)^	Hígidos	LINS	● Pior desempenho de compreensão de fala no ruído para o grupo de adultos mais velhos (30-60 anos) em relação aos mais novos (18-30 anos);
		**N=**132		● Capacidade de usar pistas espaciais não diminui em indivíduos com audição normal até os 60 anos.
		Jovens: 36 *(12-17 anos)*		
		Adultos: 96 *(18-60 anos)*		
**2**	O’Beirne et al.^(37)^	Hígidos	LPFST	● Sem diferença entre os escores obtidos na OD e OE em ambos os grupos;
		**N**=63		● Melhora no desempenho com o aumento da idade entre 17 e 34 anos, com declínio da compreensão de fala a partir dos 35 anos.
		Adultos= 15 *(28,5 anos)*		
		Crianças= 15 *(10,1 anos)*		
**3**	Liberalesso et al.^(57)^	Privação de sono	SSW T, RGD	● Pior desempenho no RGDT e SSW após 24 horas de privação de sono;
		**N**=90 *(18-40 anos)*		● Sem efeito do sexo no SSW e RGDT.
**4**	Sininger et al.^(35)^	Lateralização da audição	Discriminação de intensidade, frequência e resolução temporal	● Menor limiar para a detecção de intervalo de silêncio na OE;
		**N**=34 *(18-32 anos)*		● Vantagem da OE para estímulo tonal, sem vantagem para o ruído;
		***Escolaridade:*** Média - 15,69 anos		● Diferença entre OD e OE para a discriminação de intensidade diminuiu com a idade.
		***Treinamento musical:*** Média - 2,7 anos		
**5**	Iliadou et al.^(28)^	Psicose	GIN, RGDT	● Sem diferença entre o grupo Psicose e Músicos no teste GIN;
		**N**=90		● Grupo Psicose com melhor desempenho no GIN em relação ao RGDT;
		Psicose= 17 *(18-48 anos)*		● Melhor desempenho para o grupo de Músicos no RGDT.
		Músicos= 11 *(28-61 anos)*		
**6**	Saunders et al.^(58)^	Veteranos de guerra expostos a explosão	HINT, LISN-S, ATTR, TCST, SSW	● 75% relatou ter dificuldade de compreender a fala no ruído;
		**N**=99 *(25-53 anos)*		● 56,6% com dificuldade de acompanhar conversas;
		***Queixa:*** 80% migrânea; 73% Tontura		● 60% apresentaram alteração no HINT e 33,7% no SSW.
		***Condição de saúde:*** 19% TEPT		
		***Escolaridade*** : Ensino médio - graduação		
**7**	Prestes et al.^(29)^	Gagueira	TPD, RGDT	● Grupo gago com melhor desempenho no TPD e RGDT em relação aos não gagos, com valores inferiores à normalidade.
		**N**=41		
		Controle= 21 *(18-46 anos)*		
		Estudo= 20 *(18-46 anos)*		
**8**	Kumar et al.^(39)^	Hígido	TFC, SPIN	● Adultos mais jovens com melhor desempenho em ambos os testes;
		**N**=29		● Pior desempenho de adultos jovens e mais velhos com o aumento da taxa de compreensão e/ou da relação sinal-ruído.
		Controle= 15 *(18-25 anos)*		
		Estudo= 14 *(30-50 anos)*		
**9**	Przewoźny et al.^(59)^	Hipertensão arterial	MAA, RGDT	● Grupo Hipertensão arterial com maiores limiares de detecção de intervalo de silêncio, porém sem diferença significativa entre os grupos;
		**N**=64		● Grupo Hipertensão arterial com pior desempenho de localização sonora.
		Controle= 32 *(52,8 anos)*		
		Estudo= 32 *(53,1 anos)*		
		***Condição de saúde:*** Tratamento farmacológico; Incidência de hiperlipidemia; Tabagismo.		
**10**	Santiago et al.^(60)^	Hígidos	MLD	● Correlação positiva entre o MLD e as ondas V, A e F do FFR;
		**N**=20 (18-30 anos)		● Quanto maior a latência das ondas V, A e F, maior o MLD.
**11**	Roup et al.^(61)^	Dificuldade auditiva	SCAN-3:A, MLD, GIN, TDD, SPIN	● Todos os indivíduos com queixa apresentaram desempenho alterado em pelo menos um dos testes comportamentais;
		**N=**37		● 12% apresentaram alteração MLD e SCAN-A:3, 41% no TDD, 53% no GIN, 71% a 88%.
		Contole: 20 *(19-27 anos)*		
		Estudo: 17 *(18-58 anos)*		
**12**	Gallun et al.^(62)^	Veteranos de guerra expostos à explosão	GIN, TDD, TPF, SSW, MLD	● Veteranos de guerra e grupo controle com pior desempenho da OD no GIN;
		**N**=59		● Veteranos de guerra com pior desempenho no TDD, SSW, TPF e MLD.
		Controle= 29 *(39,2 anos)*		
		Estudo= 30 *(37,3 anos)*		
		**Condição de saúde:** 56,7% de TEPT		
**13**	Mishra et al.^(24)^	Diabetes Mellitus tipo 2	GDT	● Grupo Diabetes com maior limiar de detecção de intervalo de silêncio;
		**N**=30		● Média do limiar no GDT: GE= 6,49 ms (0,81); GC= 3,33 ms (0,79).
		Controle = 15 (30 - 40 anos)		
		Estudo = 15 (30 - 40 anos)		
**14**	Ibraheem et al.^(23)^	Zumbido	GIN	● Grupo zumbido com pior desempenho no teste GIN;
		**N**=30		● Sem correlação entre o GIN e a duração do zumbido, escala subjetiva, perfil audiológico e medidas psicoacústicas do zumbido;
		Controle = 15 (20 - 45 anos)		● Correlação positiva entre a amplitude das EOA e os escores do GIN.
		Estudo = 15 (20 - 45 anos)		
**15**	Silva et al.^(33)^	Diabetes Mellitus tipo I	Lista de Sentenças em Português	● Diferenças significantes entre os grupos com e sem diabetes para o limiar de reconhecimento no silêncio, no ruído e na relação sinal-ruído.
		**N=**40		
		Controle=20 (18-30 anos)		
		Estudo=20 (18-30 anos)		
**16**	Hoover et al.^(34)^	Lesão Cerebral Traumática Leve	QuickSIN, SRM	● Presença de *handicap auditivo* aumenta a probabilidade de pior desempenho na fala no ruído;
		**N**=33		● Sem diferença no desempenho da compreensão de fala em indivíduos com e sem lesão cerebral traumática leve.
		Controle= 9 *(18-24 anos)*		
		Estudo= 13 *(25-71 anos)*		
		Pareado= 11 *(20-70 anos)*		
**17**	Arcuri et al.^(27)^	Gagueira	TFR, TDNV, SSW, TPD, TPF, SSI, RGDT	● Grupo gago com pior desempenho nos testes TDNV e TPF;
		**N**=30		● 14 participantes do Grupo Gago apresentaram alteração do TPAC.
		Controle= 15 (18-40 anos)		
		Estudo= 15 (18-40 anos)		
**18**	Yeend et al.^(31)^	Exposição ao ruído	LISN-S, NALDCT, TFS, AM	● Sem correlação entre a exposição ao ruído ao longo da vida e o desempenho as tarefas de processamento auditivo;
		**N**=122 (30-60 anos)		● Correlação positiva entre compreensão de fala no ruído e memória de trabalho, atenção, limiares tonais de alta frequência e força de supressão do sistema olivococlear medial.
		***Queixa:*** Zumbido; dificuldade de compreensão de fala no ruído; desconforto para sons intensos		
		***Escolaridade:*** 68% Graduados; 25% Qualificação técnica; 6% Ensino médio		
		***Condições de saúde:*** Tabagismo; uso de ototóxico; história de otite		
		***Treinamento musical:*** 18% ≤ 8 anos; 40% ≥ 8 anos; 17% profissionais; 25% sem experiência		
		***Exposição a ruído:*** 70% ocupacional		
**19**	Fostick et al.^(32)^	Dislexia	Julgamento de ordem temporal	● Grupo dislexia com pior desempenho no processamento temporal;
		**N=**101		● Correlação positiva entre desempenho da memória de trabalho e processamento temporal com a leitura e processamento fonológico.
		Controle= 23 (20-33 anos)		
		Estudo= 78 (20-33 anos)		
		**Escolaridade:** 13 a 15 anos		
**20**	Habibi et al.^(25)^	Esclerose múltipla	SSW versão persa, TDD	● 46% do Grupo esclerose múltipla apresentou alteração no SSW;
		**N**=90		● Grupo esclerose múltipla com maior porcentagem de erros qualitativos e quantitativos no SSW e pior desempenho no TDD.
		Controle= 45 (25-45 anos)		
		Estudo= 45 25-45 anos)		
		**Esclerose Múltipla=** 04 a 10 anos		
**21**	Trott et al.^(20)^	Pós-menopausa	TDD, TPD, LINS-S, SPIN	● Sem diferença entre mulheres na pré e pós menopausa para TDD, TPD e SPIN-R;
		**N**=28 *(18-70 anos)*		● Pior desempenho do Grupo pós-menopausa no LISN.
		Controle= 14		
		Estudo= 14		
**22**	Sanguebuche et al.^(63)^	TPAC	DSI, MLD, TPF, TPD, RGDT, TFCA	● 18 a 29 anos: Melhores escores para o GC, exceto na OD do DSI e em ambas as orelhas do TFCA
		**N**=94		● 30 - 58 anos: Melhores escores para o GC no DSI (OE), *RGDT* e TFCA (OD).
		Controle = 64 (18-59 anos)		
		Estudo = 30 (18-59 anos)		
		***Escolaridade:*** Mínimo de 11 anos		
**23**	Turcatto et al.^(30)^	TPAC	TDD, TPF, TFC	● Melhor desempenho do grupo sem TPAC no TPF;
		**N**=40		● Desempenho semelhante entre indivíduos com e sem TPAC no TDD;
		Controle = 20 *(18-35 anos)*		● Associação entre pontuação na escala de autopercepção e a lista de monossílabos no TFC.
		Estudo = 20 *(18-35 anos*		
		***Escolaridade:*** 95% ensino superior incompleto		
**24**	Pham et al.^(64)^	Nicotina	GDT	● Menor limiar de detecção de intervalo de silêncio com uso de nicotina;
		**N**=14 *(18-27 anos)*		● Melhor desempenho da atenção seletiva com uso de nicotina.
		***Condições de saúde:*** Monitoramento de oxigenação		

**Legenda:** LINS = *Listening in Spatialized Noise-Sentences*; LPFST = *Low-pass Filtered Speech Test*; TPAC = Transtorno do Processamento Auditivo Central; OD = Orelha Direita; OE = Orelha Esquerda; SSW = *Staggered Spondaic Word Test*; TEPT = Transtorno de Estresse Pós-Traumático; RGDT = *Random Gap Detection* *Test*; ms = Milisegundos; GIN = *Gap in Noise Test*; HINT = *Hearing in Noise Test*; ATTR = *Adaptive Tests of Temporal Resolution*; TCST = *Time Compressed Speech Test*; TPD = Teste Padrão de Duração; TFC = Teste de Fala Comprimida; SPIN = *Speech Perception in Noise Test*; GC = Grupo Controle; GE = Grupo Estudo; MAA = *Horizontal Minimum Audible Angle Test*; MLD = *Masking Level Difference*; FFR = *Frequency Following Response*; SCAN A = *Tests for Auditory Processing Disorders in Adolescents* *and Adults*; TDD = Teste Dicótico de Dígitos; TPF = Teste Padrão de Frequência; GDT = *Gap Detection Threshold*; QuickSIN = *Quick Speech in Noise*; SRM = *Spatial Release from Masking*; EOA = Emissão Otoacústica; TFR = Teste de Fala com Ruído; TDNV = Teste Dicótico não Verbal; NALDCT = *National Acoustic Laboratories Dynamic Conversations Test*; TFS = *Temporal Fine Structure*; AM = *Amplitude Modulation*; SSI = *Synthetic Sentence Identification*; DSI = Identificação de Sentenças Dicóticas; TFCA = Teste de Fala Comprimida Adaptado

### Características dos estudos: tipo, população e qualidade do estudo

Na análise quanto ao tipo de estudo, identificou-se que 70,8% (17/24) eram do tipo caso controle e 29,2% (7/24) do tipo observacional.

Os estudos do tipo caso-controle (17/24) avaliaram populações diversas, com determinadas condições, a diabetes mellitus (estudos 13 e 15), a gagueira (estudos 7 e 17) e o TPAC (estudos 22 e 23), tiveram ocorrência de 11,7% (2/17); as demais condições foram privação de sono (estudo 3), psicose (estudo 5), hipertensão arterial (estudo 9), queixa de compreensão de fala (estudo 11), zumbido (estudo 14), lesão cerebral traumática leve (estudo 16), exposição ao ruído (estudo 18), dislexia (estudo 19), esclerose múltipla (estudo 20), pós-menopausa (estudo 21) e por fim, a exposição a nicotina (estudo 24), todos com ocorrência de 5,9% (1/17).

Dos estudos observacionais (7/24), 43,9% (3/7) investigou o desempenho entre as diferentes idades (estudo 1, 2 e 8), 28,6% (2/7) a população com história de exposição ao ruído (estudos 6 e 18), 14,3% (1/7) a lateralização da escuta (estudo 4) e a correlação entre testes auditivos (estudo 10).

A partir dos critérios da *Newcastle-Ottawa Scale*, 82,3% (14/17) dos estudos (3, 7, 9, 11, 12, 13, 14, 15, 17, 20, 21, 22, 23 e 24) obtiveram classificação superior a seis, indicando a qualidade dos estudos. Para os estudos observacionais, na análise pela *Quality Assessment Tool for Observational Cohort and Cross-Sectional Studies*, identificou-se que 100% apresentaram pontuação que indica boa qualidade (estudos 1, 2, 4, 6, 8, 10, 18).

### Diferenças de desempenho nos testes comportamentais em função da condição

Dentre as condições avaliadas nos estudos de caso-controle, 94,1% (16/17) identificou que o “grupo caso” apresentou pior desempenho em uma ou mais habilidades auditivas em relação ao grupo controle. Nos estudos em que mais de uma habilidade auditiva foi investigada, algumas delas diferenciam os grupos, a saber: a localização sonora para hipertensão arterial (estudo 9); a escuta dicótica, fechamento auditivo e resolução temporal no TPAC (estudo 22 e 23); figura fundo não verbal e ordenação temporal na gagueira (estudo 17); resolução temporal na psicose (estudo 5); e fechamento auditivo na pós menopausa (estudo 21). Para a condição de lesão cerebral traumática leve (estudo 16) não houve diferença entre os grupos, representando 5,9% (1/17) dos estudos.

Os critérios que conduziram à constituição do “grupo caso” são diversos e refletem condições e/ou características que já foram descritas como fatores etiológicos ou comórbidas ao TPAC. Este fato se justifica uma vez que existe uma heterogeneidade etiológica para o TPAC^(1)^, já documentada nos casos de desordens metabólicas crônicas^(24,33)^, vasculares^(59)^, desmielinizantes^(25)^, hormonais^(20)^, psiquiátricas^(28)^, do sono^(57)^, de aprendizagem^(32)^ e de fluência^(27,29)^.

Em todas as condições relatadas acima o desempenho nas habilidades auditivas foi inferior no grupo caso, principalmente para as habilidades temporais e de fechamento auditivo, validando a importância de assistir estas populações. É necessário ressaltar que duas das condições abordadas não iniciam-se na vida adulta, a dislexia^(32)^ e a gagueira do desenvolvimento^(27,29,32)^ são condições presentes desde a infância e a relação estabelecida com o PAC impacta negativamente estes indivíduos durante toda a vida^(27,29,32)^.

Outra consideração importante sobre as condições estudadas é a exposição ao ruído ser a mais explorada^(31,58,59)^. Um de seus efeitos nocivos é o dano em áreas corticais responsáveis pelo PAC^(58)^, que manifesta-se como queixa de compreensão de fala, sem alteração dos limiares auditivos^(31,61)^. O estabelecimento de uma relação entre a exposição ao ruído e a habilidade de fechamento auditivo é uma tarefa complexa, devido à influência de fatores supramodais à audição^(31)^. Entretanto, independente disto, sabe-se que esta população apresenta desempenho aquém ao esperado, sem melhora espontânea mesmo após anos sem estar exposta a ruídos de forte intensidade^(31,58,62)^. Por fim, cabe apontar a condição de exposição à nicotina^(64)^, este foi o único estudo que investigou uma possibilidade de tratamento a partir da hipótese que esta substância aumentaria a função de *gating* auditivo em situações adversas de escuta. O uso manipulado da nicotina favorece a atenção seletiva e pode ser utilizada em jovens adultos com déficits acetilcolinérgicos^(64)^.

### Diferenças de desempenho nos testes comportamentais em função da idade

Apenas 12,5% (3/24) dos estudos (1, 2 e 8) mensuraram as diferenças de desempenho em testes comportamentais do PAC ao longo da vida adulta. Todos eles avaliaram a habilidade de fechamento auditivo, com diferentes testes, sendo eles o *Listening in Spatialized Noise-Sentences* (estudo 1), *Low-pass Filtered Speech Test* (estudo 2), *Time-Compressed Speech Test* e *Speech Perception in Noise* (estudo 8). Os três estudos identificaram que adultos mais velhos apresentaram pior desempenho que os adultos mais novos. Especificamente, o estudo 1 identificou que o desempenho da compreensão de fala no ruído de adultos de 30 a 60 anos foi inferior ao de adultos de 18 a 30 anos. O estudo 2 verificou que a habilidade de fechamento auditivo se aprimora até os 34 anos e entra em declínio a partir desta idade. O estudo 8 identificou que adultos de 18 a 25 anos apresentaram melhor desempenho nos dois testes aplicados em comparação aos adultos de 30 a 50 anos.

A partir destes resultados evidencia-se que poucos estudos dedicam-se à investigação do PAC em relação às mudanças inerentes ao aumento da idade na vida adulta^(36,37,39)^. Todos eles avaliaram apenas a habilidade de fechamento auditivo. Independente do tipo de estímulo empregado, palavras ou frases, os achados entre os estudos foram semelhantes, indicando que adultos com menos de 60 anos apresentam desempenho inferior ao de jovens adultos. A partir destes estudos pode-se inferir que adultos com mais de 30 anos experienciam desvantagens em condições adversas de escuta, mesmo que a capacidade de análise de pistas acústicas dos estímulos sonoros não sofra este declínio^(36,37,39)^.

Este conhecimento sustenta a importância da investigação dos distúrbios auditivos em nível do SNAC da população adulta saudável, incluindo todas as habilidades auditivas e comparando grupos com menor variação de idade. Isto permitiria a compreensão deste processo dinâmico de aumento da idade tanto em função, quanto em tempo de início.

Embora seja um desafio identificar em que momento da vida adulta inicia-se o declínio do PAC, para cada um dos mecanismos e habilidades, esta investigação é necessária, uma vez que processamento de informações em tempo hábil é essencial para comunicação e a desaceleração relacionada à idade está bem documentada nos domínios cognitivos e sensoriais.

### Caracterização do população estudada

Um único estudo (4,2%) caracterizou a população investigada em função de variáveis independentes, a condição de saúde e a exposição ao ruído ocupacional e de lazer (estudo 18). A condição de saúde foi caracterizada quanto ao uso de medicamentos potencialmente ototóxicos, contato com ototóxicos no geral, história de tabagismo, infecção de ouvido e zumbido. Outros estudos fizeram algum tipo de caracterização, mas não as analisaram como variável independente para a condição investigada.

### Investigação comportamental do processamento auditivo

A partir dos 24 estudos incluídos foi possível identificar mais de 20 variações de testes comportamentais. Destes o mais aplicado foi o *Random Gap Detection Test* (RGDT) presente em 25% (6/24) dos estudos, seguido dos Testes Padrão de Frequência (TPF), Dicótico de Dígitos (TDD) e *Staggered Spondaic Word* (SSW) em 20,8% (5/24) dos estudos. Os testes *Listening in Spatialized Noise-Sentences*, *Gap in Noise*, Padrão de Duração e *Masking Level Difference* foram realizados em 16,6% (4/24) dos estudos. Os outros testes foram aplicados em três ou menos estudos.

Dentre as habilidades auditivas avaliadas, a de fechamento auditivo foi a mais investigada (54,1%; 13/24), seguida das habilidades de resolução (45,8%; 11/24) e ordenação temporal (41,6%; 10/24). As habilidades de figura-fundo verbal e interação binaural foram avaliadas em 29,1% (7/24) dos estudos e a de figura-fundo não verbal em 4,1% (1/24). Apenas 20,8% (5/24) dos estudos avaliaram exclusivamente uma única habilidade auditiva, 12,5% (3/24) resolução temporal e fechamento auditivo e 4,1% interação binaural e figura-fundo verbal.

Embora os testes que constam em um maior número de artigos sejam o RGDT, TPF, TDD e SSW, os testes de baixa redundância foram identificados em maior diversidade, mais de 10 testes eram destinados à avaliação da habilidade de fechamento auditivo. Este achado deve ser discutido quanto a esta ser a habilidade auditiva mais investigada, possivelmente por ser intrinsecamente relacionada à compreensão de fala^(14,52,53)^, e quanto ao grande número de testes encontrados, provavelmente pelas características que estes devem possuir. Estes devem ser validados no idioma da população avaliada e os parâmetros de gravação como frequência, ressonância e modulação vocal, articulação e velocidade de fala devem ser o mais adequados e naturais possíveis^(37)^. Deve-se considerar também a escolha do material de fala e das redundâncias intrínsecas, seja por estímulo competitivo ou pela degradação do estímulo, e da localização da fonte sonora. Estas características tornam o desenvolvimento e a escolha destes testes um desafio, pois a tentativa é que se aproximem ao máximo das situações adversas de escuta presentes no dia-a-dia^(37,65)^.

### Determinação da condição da habilidade auditiva e do processamento auditivo

Em relação a utilização de valores normativos, 33,3% (8/24) indicaram o uso de referências, destinadas à população adulta, para classificar o desempenho nos testes comportamentais como adequado ou alterado (estudos 3, 7, 9, 11, 16, 17, 22 e 23) . Dois dos 24 estudos identificados (8,3%) tiveram como objetivo determinar a presença de TPAC (estudos 22 e 23), para isto os critérios utilizados foram a alteração em uma ou mais habilidades auditivas^(52)^ (estudo 22) e alteração no testes dicótico de dígitos e/ou padrão de frequência (estudo 23).

A aplicação e interpretação dos testes segundo as recomendações de produção e/ou padronização reduzem a variabilidade de interpretações e aumentam o consenso clínico quanto aos resultados^(66)^. O diagnóstico do TPAC foi objetivo de dois estudos, entretanto apenas um deles o fez conforme recomendação de especialistas da área^(63)^. É bem estabelecido na literatura que a avaliação diagnóstica do TPAC deve ser realizada por meio de diferentes testes comportamentais que sejam sensíveis e específicos para identificar disfunções no SNAC^(1,2,14,52,53)^.

### Investigação complementar

A aplicação de testes complementares a avaliação do PAC foi realizada em 58,3% (14/24) dos estudos. Destes 14 estudos, 28,6% (4/14) aplicaram testes eletrofisiológicos auditivos, sendo 75% (3/4) o PEATE clique (estudos 10, 21 e 22) e 25% (1/4) o FFR (estudo 10), o PEAML (estudo 21) e o PEALL (estudo 7). O uso de emissões otoacústicas ocorreu em 35,7% (5/14) dos estudos, variando entre produto de distorção (estudos 4, 9 e 18) e transiente (estudos 14 e 17). Quanto à autopercepção, 50% (7/24) dos estudos aplicaram questionários buscando caracterizar a percepção dos participantes sobre a função auditiva (estudos 6, 11, 12, 14, 16, 18 e 23). O estado de consciência mental, em forma de rastreio e avaliação, foi investigado em apenas 21,4% (3/24) dos estudos (6, 12 e 18).

Os testes aplicados como complementares podem auxiliar no diagnóstico do TPAC, bem como, na delimitação desta população tipicamente heterogênea^(1,2)^, entretanto a presente revisão identificou que em estudos com adultos esta não é uma prática comum. Os questionários de autopercepção foram a forma de avaliação complementar mais aplicada, possivelmente porque alguns questionários apresentaram correlação significativa com os achados dos testes comportamentais auditivos^(1,2,53)^. Os testes objetivos, eletrofisiológicos e eletroacústicos, foram aplicados em poucos estudos. A literatura preconiza que estes sejam incluídos na avaliação do PAC, uma vez que viabilizam a avaliação da integridade funcional e estrutural da via auditiva e ampliam a compreensão dos achados dos testes comportamentais^(1,14,53)^. Por fim, o rastreio do estado mental foi a avaliação complementar menos realizada, esta garante que os achados referentes ao PAC não são consequências de alterações cognitivas significativas, excluindo este fator preditor. A partir disto, cabe a reflexão de que estes fatores que delimitam a população e auxiliam no diagnóstico devem ser empregados devido a heterogeneidade do TPAC e a influência de fatores supramodais na audição na avaliação comportamental.

## CONCLUSÃO

A maior parte dos estudos elegíveis objetivou a avaliação de determinado mecanismo e/ou tarefa auditiva em populações específicas, e não o diagnóstico do TPAC em si. O teste mais utilizado foi o *RGDT,* enquanto que a habilidade de fechamento auditivo foi a mais investigada, com maior diversidade de testes. Também foi identificada uma heterogeneidade na população estudada, quanto à característica para formação dos grupos caso. As formas de avaliação complementar foram testes eletrofisiológicos, eletroacústicos, questionários de autopercepção e rastreio do estado mental.
